# Comparison of caffeine consumption behavior with plasma caffeine levels as exposure measures in drug-target mendelian randomization

**DOI:** 10.1093/aje/kwae143

**Published:** 2024-06-20

**Authors:** Benjamin Woolf, Héléne T Cronjé, Loukas Zagkos, Susanna C Larsson, Dipender Gill, Stephen Burgess

**Affiliations:** School of Psychological Science, University of Bristol, Bristol, UK; MRC Integrative Epidemiology Unit, University of Bristol, Bristol, UK; MRC Biostatistics Unit at the University of Cambridge, Cambridge, UK; Department of Public Health, Section of Epidemiology, University of Copenhagen, Copenhagen, Denmark; Department of Epidemiology and Biostatistics, School of Public Health, Imperial College London, London SW7 2BX, United Kingdom; Unit of Medical Epidemiology, Department of Surgical Sciences, Uppsala University, Uppsala, Sweden; Unit of Cardiovascular and Nutritional Epidemiology, Institute of Environmental Medicine Karolinska Institutet, Stockholm, Sweden; Department of Epidemiology and Biostatistics, School of Public Health, Imperial College London, London SW7 2BX, United Kingdom; MRC Biostatistics Unit at the University of Cambridge, Cambridge, UK; Cardiovascular Epidemiology Unit, Department of Public Health and Primary Care, University of Cambridge, Cambridge, UK

**Keywords:** instrument validity, instrument selection, caffeine metabolism, pharmacoepidemiology, causal inference, genetic epidemiology, drug target Mendelian randomization

## Abstract

Mendelian randomization is an epidemiologic technique that can explore the potential effect of perturbing a pharmacological target. Plasma caffeine levels can be used as a biomarker to measure the pharmacological effects of caffeine. Alternatively, this can be assessed using a behavioral proxy, such as average number of caffeinated drinks consumed per day. Either variable can be used as the exposure in a Mendelian randomization investigation, and to select which genetic variants to use as instrumental variables. Another possibility is to choose variants in gene regions with known biological relevance to caffeine level regulation. These choices affect the causal question that is being addressed by the analysis, and the validity of the analysis assumptions. Further, even when using the same genetic variants, the sign of Mendelian randomization estimates (positive or negative) can change depending on the choice of exposure. Some genetic variants that decrease caffeine metabolism associate with higher levels of plasma caffeine, but lower levels of caffeine consumption, as individuals with these variants require less caffeine consumption for the same physiological effect. We explore Mendelian randomization estimates for the effect of caffeine on body mass index, and discuss implications for variant and exposure choice in drug target Mendelian randomization investigations.

## Introduction

Mendelian randomization (MR) is a popular epidemiologic technique for exploring potential causal effects using observational data.^1^^,^^2^ In analogy with a randomized controlled trial, MR leverages random variation in the inheritance of genetic variants at conception to improve the robustness of epidemiologic findings to reverse causation and confounding. The implementation of MR using an instrumental variables framework has been facilitated by the availability of genome-wide association study (GWAS) summary statistics.^3^^,^^4^

Drug-target MR applies this technique to explore the potential effect of intervening on a pharmacological target.^5–7^ In drug-target MR, it is typical to select variants from within a gene region which codes for a biomarker that reflects perturbation of the target of interest.^6^ While this variant selection strategy typically means the MR assumptions are more plausible, it requires understanding of the underlying biology of the drug target.

Studies like UK Biobank have measured participants’ self-reported exposure to many pharmacological compounds,^8^ such as medication use. These data enable an alternative MR strategy, in which genome-wide significant variants associated with the target’s behavioral proxy are used instead of biologically relevant variants.^9^ Since these phenotypes are generally cheaper to measure than biomarker or protein expression data, they may allow for larger sample sizes in analyses.^10^

Caffeine consumption behavior (eg average number of cups of coffee or tea consumed in a typical day) is a popular exposure for studying the effects of increased exposure to caffeine.^11–16^ MR studies using plasma caffeine levels as the exposure as predicted by variants known to affect the metabolism of caffeine have produced opposing findings to MR studies using variants associated with self-reported caffeine consumption. For example, observational studies have found that greater exposure to caffeine is predictive of lower body mass index (BMI) and type 2 diabetes mellitus risk.^11^^,^^12^^,^^17^ Likewise, randomized controlled trials indicate that caffeine intake may promote weight, BMI, and body fat reduction.^18^ MR studies using genome-wide significant variants for caffeine consumption have failed to replicate the inverse associations of caffeine consumption with BMI and type 2 diabetes risk.^11–13^ Larsson et al., however, found results consistent with the observational and trial literature when using variants with biological relevance to plasma caffeine levels.^19^ They selected the lead variant associated with plasma caffeine levels from the Cytochrome P450 Family 1 Subfamily A Member 2 (*CYP1A2)* and Aryl Hydrocarbon Receptor (*AHR)* gene regions which are known to impact the metabolism of caffeine.

A caffeine consumption GWAS could be used to study the effect of caffeinated drink consumption in an MR study. However, there may be effects of caffeinated drink consumption other than through caffeine levels. The relevant question from a drug development perspective is to assess the effects of perturbing the pharmacological target (here, caffeine levels). We focus on how the genetic architecture of caffeine consumption behavior relates to that of plasma caffeine levels. We will use this to highlight two potential limitations of using behavioral proxies for understanding the effect of perturbing a pharmacological target (caffeine levels): misidentification of the true exposure, and increased risk of invalid instruments due to pleiotropy.

## Methods

### Study overview

**Figure 1:**
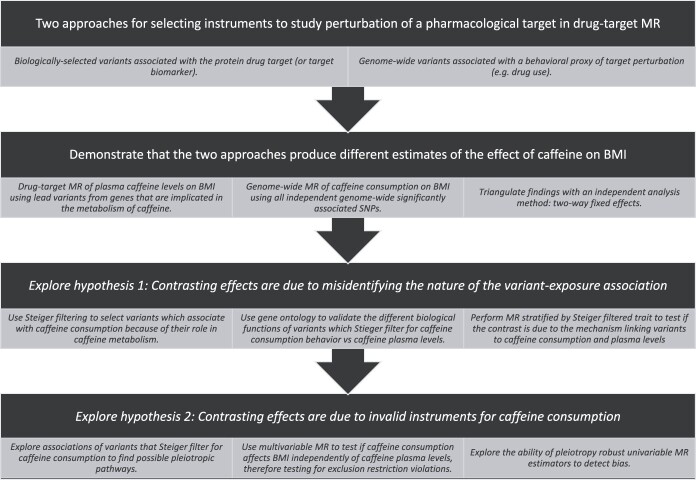
Study overview figure.

We aim to highlight two possible issues arising from using a behavioral proxy of target perturbation in a MR study where the goal is validation of the pharmacological target: 1) misidentifying the direction of the effect by not accounting for the mechanism causing the variant-exposure association, and 2) increased risk of including invalid instruments (Figure 1). First, we reproduce the contrasting effects of caffeine on BMI using genome-wide and biologically-selected instruments. We then use a different quasi-experimental method, two-way fixed effects, to adjudicate between results. Second, we explore the mechanism underlying variant-caffeine consumption associations and the extent to which this accounts for the contrasting effects. Finally, we explore if the use of behavioral phenotypes for variant selection could induce violations of the instrumental variable assumptions.

### Assessing the effects of caffeine on body mass index

#### Genome-wide mendelian randomization analyses for caffeine consumption

We selected single nucleotide polymorphisms (SNPs) associated with self-reported coffee consumption in the UK Biobank. As a supplementary analysis, we also extracted SNPs associated with self-reported tea consumption. These GWASs were conducted according to a standardized pipeline described elsewhere.^20^ To have sufficient power to detect an effect, we selected uncorrelated (clumping r^2^ = 0.001) variants using a *P* value threshold of 5 ×10^-7^. Variant-outcome associations were taken from the 2018 GIANT Consortia meta-analysis of BMI, comprising 681 275 participants.^21^ Variant-trait data were extracted from the OpenGWAS platform using the UK Biobank phenotype IDs: ukb-b-6066, ukb-b-5237, and ieu-b-40.^22^ We used the TwoSampleMR R package to harmonize the results, and combined SNP data using the inverse-variance weighted method.^23^

### Biologically-motivated mendelian randomization analysis for plasma caffeine levels

We used the lead variants within the *CYP1A2* and *AHR* gene regions (rs2472297 and rs4410790 respectively). Summary data on the association of these variants with fasting plasma caffeine levels were retrieved from Cornelis et al..^24^ This was a meta-analysis of 6 studies, including 9876 European ancestry participants. Variant-outcome data were taken from the same GIANT meta-analysis. This MR analysis was otherwise conducted using identical methods to the caffeine consumption analysis.

#### Triangulation with two-way fixed effects

Confidence in a study’s results can be strengthened by triangulating with an alternative design which makes different assumptions.^25^ One such design is two-way fixed effects (TWFE), as described in Appendix 1.^26–29^ We note that the estimands from the approaches are likely to differ (one reason being that MR estimates typically reflect lifelong differences in the exposure distribution, whereas TWFE estimates represent shorter term differences). However, if the assumptions are satisfied, the direction of estimates from the approaches should be consistent.

We implemented the TWFE model using the fixest R package, and accounted for clustering from both fixed effects in the standard errors.^30^ Specifically, the UKB research team asked participants to report the number of cups of tea and coffee they drank in a typical day (UKB phenotype IDs: 1488 and 1498) at all four assessment center visits (501 472 participants at recruitment, 20334 at the first repeat assessment visit, 64 924 at the first imaging visit, and 5360 at the repeat imaging visit). We standardized the number of cups of tea and coffee measures at each time point and combined them to create a measure of caffeine consumption. One standard deviation of caffeine consumption in the UKB equates to around 2 cups of coffee a day or three cups of tea. At each of these visits, the BMI of each participant was also calculated (UKB phenotype ID: 21001).^8^^,^^31^

### Exploring the genetic architecture of caffeine consumption behavior

One well-measured behavioral phenotype studied using MR is smoking. The genetics of cigarette smoking can be split between variants which affect smoking initiation, and those which impact on smoking heaviness.^32^^,^^33^ Variants in the nicotinic receptor genes inhibit the metabolism of nicotine. While variants in this gene are not strong predictors of smoking initiation, people who smoke and carry certain variants associated with reduced metabolic inhibition smoke more than those without these variants to get the same physiological nicotine effect. For example, each copy of the rs16969968 minor allele is associated with smoking one additional cigarette per day among current smokers.^34^ Thus, an MR study exploring the effects of nicotine using instruments from a GWAS of smoking heaviness could provide counterintuitive estimates, since people who smoke more would have lower genetically-predicted nicotine levels.

This vignette closely parallels the explanation provided by Larsson et al. for the discordant estimates between their study using plasma caffeine as the exposure,^19^ and studies using caffeine consumption phenotypes. They found that variants in the *CYP1A2* and *AHR* genes both predict *greater* plasma caffeine levels and consumption of *less* caffeine in UK Biobank. Since over 95% of UK Biobank participants drank caffeine regularly, one possibility is that the variants associated with higher caffeine consumption are reflective of the need to consume more caffeine for the same effect due to increased metabolic efficiency (ie a shorter caffeine half-life). Analogous to the example of nicotine, it may be that people who consume more caffeine do so because they have lower circulating caffeine levels. Since caffeine is not produced endogenously, the opposite conclusion (that caffeine consumption reduces circulating caffeine plasma levels) is biologically implausible.

### Using Steiger filtering to identify metabolism and behaviorally mediated caffeine consumption SNPs

For the above hypothesis to explain the discrepancies in the results, most SNPs which associate with caffeine consumption behavior should do so because of their effect on caffeine metabolism. Generally speaking, the more distal an outcome is from a cause, the less variance the cause will explain in the outcome. Steiger filtering is a statistical method that leverages this principle to determine which trait is the proximal effect of a genetic variant by comparing the variance explained by the variant in the traits.^35^ In its simplest implementation, a SNP will Steiger filter for one trait over a second when it explains a greater percentage of the variation in the first trait than the second. We used Steiger filtering to explore which variants are acting on caffeine consumption via caffeine metabolism (analogous to smoking heaviness), and which are primarily acting through other pathways. As a supplementary positive control, we used Steiger filtering to confirm that the *CYP1A2* and *AHR* variants metabolically affect circulating caffeine levels before affecting downstream caffeine consumption behavior.

#### Validating Steiger filtering using gene ontology.

To support the biological validity of the conclusions drawn from the Steiger filtering, we explore if the two sets of SNPs have different ontologies. After mapping each variant to its genomic locus, we used the gene ontology database^36^ to compare gene overrepresentation between the plasma levels-associated and behavior-associated variants (FDR-adjusted Fisher’s exact tests, PANTHER 17.0, http://pantherdb.org/).

#### SNP mechanism stratified MR to explore exposure misidentification

If the SNPs which Steiger filter for caffeine plasma levels associate with caffeine consumption because of their role in caffeine metabolism, then we would expect the contrasting effects to be explained by an inverse effect of caffeine levels on caffeine consumption. We therefore use MR to estimate associations of genetically-predicted caffeine plasma levels with caffeine consumption for the SNPs which Steiger filtered for each of the respective traits. We also repeat the genome-wide caffeine consumption MR analysis (weighted by both caffeine consumption and caffeine plasma levels) on BMI, stratifying the analysis by which trait the SNPs Steiger filtered for.

### Exploring if caffeine consumption variants are invalid instruments

#### Exploring pleiotropic pathways in behaviorally mediated caffeine consumption SNPs

**Figure 2:**
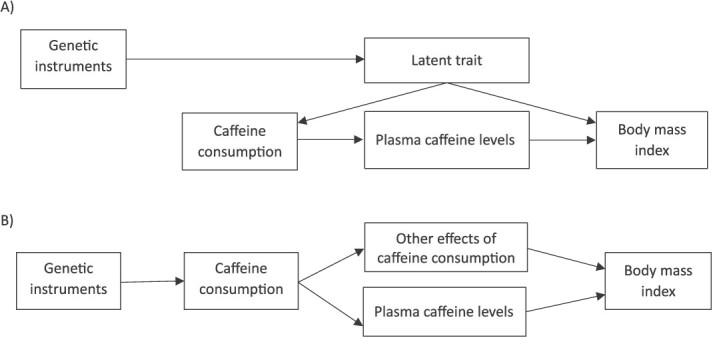
Directed acyclic graphs (DAGs) of potential exclusion restriction violations when using caffeine consumption as an exposure phenotype for MR studies exploring the effects of exposure to greater plasma caffeine levels. Pannel a: Exclusion restriction violations due to instrumenting an underlying latent trait, eg health seeking behavior and medication use. Pannel B: Exclusion restriction violations because of other effects of caffeine consumption which are not via caffeine plasma levels. This includes other metabolites consumed with caffeine, eg sugar and milk. N.B. If the exposure of interest is the total effect of caffeine *consumption*, rather than the specific effect of caffeine itself, then Pannel B does not depict a violation of the exclusion restriction assumption.

SNPs acting on behavioral intermediaries could be at greater risk of violating the exclusion restriction assumption than those directly affecting a circulating biomarker. Figure 2 presents two Directed Acyclic Graphs (DAGs) showing plausible mechanisms by which this assumption may be violated for a drug-target MR analysis. In Figure 2(A), the variants associate with caffeine consumption through an underlying latent trait which causes both consumption and the outcome, eg people that weigh less might consume fewer caffeinated drinks because they lead a healthier lifestyle. In Figure 2(B), because caffeinated drinks contain more than just caffeine, there may be an effect of these other substances (eg hot water, milk, sugar, or an accompanying snack) on BMI, even if we are correctly instrumenting caffeine consumption. Thus, an MR study trying to explore only the effect of caffeine levels could be biased by effects of these other substances.

The applicability of DAGs such as these can be supported by showing that the SNPs which Steiger filter for caffeine consumption are associated with relevant behavioral or lifestyle traits. We use PhenoScanner to explore what traits with these SNPs are associated with (at *P* < 5 ×10^-5^, p_FDR_ < 0.05).^37^

#### Multivariable MR to test for exclusion restriction violations in the caffeine consumption MR.

If the genetic instruments for caffeine consumption act entirely through the circulating bioactive caffeine metabolite, then there should be no direct effect of caffeine consumption on BMI independent of plasma caffeine levels. Conversely, finding an effect of caffeine consumption independent of the bioactive caffeine metabolite would support the existence of exclusion restriction violating pathways like those depicted in Figure 2. We use multivariable MR (MVMR) to test if there is an association of the caffeine consumption variants with BMI independent of caffeine plasma levels.

MVMR estimates the direct effect of one exposure conditional on another.^38^ Since we have access to GWAS data on caffeine plasma levels, we can empirically test if caffeine consumption traits act on BMI only through caffeine plasma levels. We selected SNPs associated (*P* < 5 ×10^-7^) with caffeine consumption or circulating caffeine levels. We then ranked these SNPs in order of their *P* values and clumped them to an r^2^ of 0.001. To minimize the effects of conditional weak instrument bias, we implemented the analysis using the MVMR-Qhet estimator.^39^

#### Univariable pleiotropy robust estimators

Both biases depicted in Figure 2 could be described as pleiotropy, but many pleiotropy robust methods, like MR Egger or weighted median, are likely to produce incorrect estimates in this context. Biases like those in Figure 2(A) will violate the MR-Egger InSIDE assumption,^40^ while those in Figure 2(B) are likely to result in a similar bias across many SNPs which would bias most estimators.^41^^,^^42^ We explore the ability of commonly used pleiotropy robust estimators (MR-Egger, weighted median, weighted mode, and simple mode^3^) to detect pleiotropy among a) all SNPs associated with coffee consumption, and b) only SNPs which Steiger filter for caffeine consumption.

## Results

### Contrasting mendelian randomization estimates for the effect of caffeine on BMI

In our genome-wide MR analysis using self-reported caffeine consumption to select variants, each standard deviation (SD) increase in the genetically-predicted amount of coffee consumed was associated with 0.754 (95% CI: 0.284, 1.224) SD higher BMI. In our biologically-motivated MR study instrumenting plasma caffeine levels, each SD increase in genetically-predicted plasma caffeine levels associated with a 0.085 SD decrease (95% CI, -0.095 to -0.075) in BMI. Under the monotonicity assumption, these MR estimates represent the average lifetime effect of exposure to higher caffeine levels among people for whom the variants influence caffeine levels (ie compliers).

The TWFE regression model found that each SD increase in caffeine consumption was associated with 0.100 (95% CI: -0.152 to -0.048) SD *lower* BMI. This therefore supports the conclusion of the biologically-motivated MR analysis.

Implications of the genetic architecture of caffeine consumption on Mendelian randomization estimates.

Steiger filtering variants associated with caffeine consumption behavior implied that 16 of the 24 coffee-consumption-associated SNPs are affecting caffeine consumption behavior because of their effect on caffeine metabolism. This interpretation was supported by the gene ontology analysis. We found that plasma caffeine level-filtered variants were significantly enriched for the biological process *response to organic substance* (3.43-fold enrichment, *P* = 8.67 ×10^-7^). However, no statistically significant gene enrichment was observed for consumption-filtered variants. This supports there being a less directly biological (eg a behavioral) mechanism linking these other variants to caffeine consumption. Our positive control analysis for Steiger filtering confirmed that the lead variants in the *CYP1A2* and *AHR* genes influence circulating caffeine levels (r^2^ = 1.4% for both SNPs combined) before caffeine consumption behavior (r^2^ = 0.2% for both SNPs combined).

The SNPs which Steiger filtered for caffeine plasma levels imply a negative MR association between circulating plasma caffeine levels and caffeine consumption (β = -0.295 SD consumed per SD increase in plasma caffeine levels, 95% CI: -0.403 to -0.187), replicating the observation by Larsson et al. Thus, although using these SNPs to estimate the effect of caffeine consumption on BMI produces a positive MR estimate (β = 0.586 SD per SD increase in coffee consumption, 95% CI: 0.047, 1.125), subsequent scaling by plasma caffeine levels results in similar MR estimates (β = -0.141 SD per SD increase in plasma caffeine levels, 95% CI: -0.353 to 0.071) to those observed in the biologically-motivated MR analysis above.

The remaining SNPs are more proximal to caffeine consumption behavior than plasma caffeine levels. The MR analysis of caffeine consumption on BMI using these SNPs still implies that increased caffeine consumption may increase BMI (β = 1.541 SD per SD increase in coffee consumption, 95% CI: 0.610, 2.472). This cannot be explained by the effect these SNPs have on circulating caffeine: MR of caffeine consumption on plasma caffeine levels are indicative of a positive direction of effect (β = 0.416 SD per SD increase in coffee consumption, 95% CI: 0.110, 0.722). A potential explanation is the increase in consumption of milk, sugar and cookies that often accompany caffeine consumption.

### Caffeine consumption variants can be invalid instruments

Many of the SNPs which Steiger filtered for caffeine consumption are associated (*P* < 5 ×10^-5^) with behavioral traits such as smoking, alcohol consumption, education, and physical activity (and BMI related traits) in PhenoScanner (Supplementary Tables S1a and S1b). Any of these could be a source of exclusion restriction violations.

In our MVMR model, we find evidence of a direct effect of coffee consumption, independent of plasma caffeine levels, on BMI. Each SD increase in coffee consumption results in a 0.556 (95% CI: 0.296, 1.829) SD increase in BMI, independent of plasma caffeine levels. This indicates that the totality of the effect on BMI is not explained by plasma caffeine levels.

Despite MVMR implying the existence of exclusion restriction violations, the traditional pleiotropy robust methods generally imply a positive causal effect of caffeine consumption on BMI when using SNPs Steiger filtered for caffeine drinking behavior (Table 1 and Supplementary Table S2).

**Table 1:** Summary and interpretation of the study’s results.

**Analysis**	**Exposure, units**	**Outcome, units**	**Variant-exposure F statistic**	**Number of SNPS**	**Estimate (95% CI)**	**Interpretation**
Genome-wide MR	Coffee consumption (SD)	BMI (SD)	239	24	0.754 (0.284 , 1.224)	Greater caffeine consumption results in weight gain.
Biologically-motivated MR	Plasma caffeine levels (SD)	BMI (SD)	71	2	-0.085 (-0.095 to -0.075)	Exposure to more caffeine results in weight loss. Since TWFE and MR make different assumptions, the triangulation between the two estimates supports the validity of the biologically-motivated MR analysis.
Two-way Fixed effects (TWFE)	Caffeine consumption (SD)	BMI (SD)			-0.100 (-0.152 to -0.048)	
Genome-wide MR using SNPs which Steiger filter for caffeine plasma levels	Plasma caffeine levels (SD)	Coffee consumption (SD)	11	16	-0.295 (-0.403 to -0.187)	Caffeine consumption SNPs which Steiger filtered for caffeine plasma levels imply a positive effect of caffeine consumption on BMI. However, when scaled by plasma caffeine levels they produce similar estimates to the biologically-motivated MR analysis. Since caffeine consumption cannot reduce caffeine plasma levels, it is likely that these SNPs measure the effect that caffeine metabolism has on caffeine consumption.
	Coffee consumption (SD)	BMI (SD)	112	16	0.586 (0.047 , 1.125)	
	Plasma caffeine levels (SD)	BMI (SD)	11	16	-0.141 (-0.353 , 0.071)	
Genome-wide MR using only SNPs which Steiger filter for caffeine consumption	Coffee consumption (SD)	BMI (SD)	42	8	1.541 (0.610 , 2.472	SNPs which Steiger filtered for caffeine consumption imply a positive effect of caffeine consumption on BMI. Since they also have a positive effect on caffeine plasma levels, this cannot be explained by misidentification of the exposure. However, these SNPs do associate with behavioral phenotypes which could also affect BMI (Supplementary Table 1)(available at https://doi.org/10.1093/aje/kwae143).
	Coffee consumption (SD)	Plasma caffeine levels (SD)	42	8	0.416 (0.110 , 0.722	
Multivariable MR	Direct effect of caffeine consumption (SD) after adjusting for plasma caffeine levels	BMI (SD)	5 (plasma caffeine) and 42 (caffeine consumption)	25	0.556 (0.296 , 1.829)	Caffeine consumption has a direct effect on BMI, independent of caffeine plasma levels. This implies that drug target MR using caffeine consumption SNPs to study the effects of plasma caffeine levels could suffer from exclusion restriction violations.
Genome-wide MR using pleiotropy robust estimators	Coffee consumption (SD)	BMI (SD)	239	24	Generally positive (Supplementary Table 2)	Overreliance in pleiotropy robust method may be inappropriate in the presence of systematic exclusion restriction violations.

Results of the supplementary analysis using data on tea consumption are similar to those using the GWAS of coffee consumption (see Appendix 2).

## Discussion

In this paper, we found evidence to support two hypotheses for the differences observed between biologically-motivated MR estimates for the effect of caffeine plasma levels on BMI, and genome-wide MR estimates for the effect of caffeine consumption on BMI. Specifically, we explored if the caffeine consumption variants are misidentifying the true exposure, and if variants selected by these approaches are valid instruments for judging the effect of plasma caffeine levels.

To explore the first hypothesis, we performed Steiger filtering to identify which SNPs associate primarily with caffeine plasma levels or caffeine consumption. Two-thirds of the SNPs for coffee consumption appear to affect caffeine consumption because of their effect on caffeine metabolism. Counterintuitively, we observed a negative MR association between genetically predicted caffeine plasma levels and caffeine consumption (Table 1). Since caffeine consumption cannot cause lower caffeine plasma levels, this is likely due to people with elevated genetically predicted plasma caffeine needing to drink less coffee or tea to experience the same physiological effect. Indeed, gene enrichment analysis revealed overrepresentation of genes involved in the metabolic response to the presence of organic substances, specifically processes resulting in physiological tolerance to an organic substance, among these SNPs. When scaling the caffeine consumption MR estimate using metabolism-related SNPs by the SNP effect on caffeine plasma levels, we find a similar association to that in the biologically-motivated MR analysis. This means that a naive interpretation of MR using caffeine consumption to proxy caffeine plasma levels may produce misleading results and demonstrates the importance of understanding the biological mechanism linking a behavioral phenotype to the drug-target biomarker.

The MR analyses of caffeine consumption on both BMI and plasma caffeine using SNPs which Steiger filtered for consumption behavior finds a positive association with BMI (Table 1). This is likely not due to the misidentification of caffeine metabolism variants as caffeine consumption variants because the direction of effect is identical in both MR analyses. Instead, we argue that these remaining SNPs are likely to be invalid instruments. This hypothesis is supported by the range of behavioral phenotypes these SNPs are associated with in PhenoScanner. Indeed, we were able to demonstrate the existence of a causal effect on BMI independent of caffeine plasma levels using MVMR. This implies that a drug-target MR using these variants would suffer from an exclusion restriction violation. Thus, SNP validity, in addition to specification of the correct exposure, can complicate the interpretation of the caffeine consumption MR effect estimates.

Finally, we triangulated with two-way fixed effects to test whether the direction of the biologically-motivated MR estimate is reliable. Since the TWFE assumption of no non-linear time varying confounding is different to the MR assumption of no pleiotropy, the negative effect in the TWFE analysis lends extra credence to the finding that increased caffeine levels result in weight loss. However, we acknowledge it is possible some factors may both violate MR and TWFE analyses, such as feedback loops between caffeine consumption and BMI.

The failure of commonly used pleiotropy robust methods to produce estimates with the correct direction of effect demonstrates the difficulty in detecting systematic pleiotropy in settings with complex behavioral exposures. These estimators typically assume that each SNP has an idiosyncratic pleiotropic effect. Researchers should not overinterpret findings in settings where similar exclusion restriction violations may affect a large proportion of SNPs. Positive and negative controls can be used to detect if related traits could result in violations of the exclusion restriction assumption.^9^ One potential control variable for caffeine consumption is green tea consumption, as green tea is typically consumed without milk and sugar. Alternatively, decaffeinated coffee consumption could be investigated to attempt to separate the biological effect of caffeine from the effect of substances typically added to hot drinks, such as milk and sugar. Currently, however, available GWASs of these traits are inadequately powered.^43–45^

Our study has assumed that the research question of interest is the effect of plasma caffeine levels, rather than caffeine consumption. A recent MR study found an association between genetically proxied coffee consumption and esophageal cancer, but no association with other cancer types.^15^ This was proposed as being the effect of consuming hot liquids. While Figure 2(B) is a description of this interpretation, this effect should not be described as an exclusion restriction violation in this case. In such a study, the behavior, rather than the drug target, is the exposure of interest. As such, the relative merits of different study designs depend on the research question being answered. When the mechanism linking metabolism and consumption behavior is understood, there may still be utility in using biologically-motivated variant selection strategies to explore the effect of consumption behavior because they might avoid biases due to pleiotropy.

We have not explored all issues with using a pharmacological target’s behavioral proxy as an exposure. It is well established that psychosocial and behavioral GWASs, such as those for drug use, are more prone to residual confounding from population structure, assortative mating, or dynastic effects than biomedical GWASs, such as biomarker levels.^46^ MR studies using a behavioral exposure to proxy pharmacological interventions may additionally suffer from confounding by indication.^47^ Therefore, the independence assumption can be less plausible when using a behavioral proxy like caffeine consumption as an exposure than when using a biomarker. Likewise, the gene–environment equivalence assumption, which is required for MR estimates to translate to the effects of pharmacological interventions, is less plausible when using these behavioral phenotypes.^48^^,^^49^

We believe that the two issues highlighted here could be relevant for other pharmacological targets. A target’s behavioral proxy can be influenced by many heritable factors, such as health seeking behavior and education. Variants selected based on behavioral proxies are therefore at greater risk of being invalid instruments. The relationship between genetic variants and caffeine phenotypes is comparatively simple and well understood when compared to other pharmacological targets. Caution is therefore required when interpreting MR studies which proxy a pharmacological target using a behavioral exposure, like drug use or vitamin supplementation, without an understanding of the underlying biology. Careful thought is always required when choosing instruments and exposure traits for drug-target MR studies.^50^ However, we believe that our results support a preference for using objectively measured biomarkers as exposures rather than behavioral proxies.

### Ethics approval statement

UK Biobank received ethics approval from the North West Multi-Centre Research Ethics Committee (REC reference 11/NW/0382). All participants provided written informed consent to participate in the study. Data from the UKB are fully anonymized.

## Acknowledgments

*This work was carried out using the computational facilities of the Advanced Computing Research Centre, University of Bristol—*  http://www.bris.ac.uk/acrc/*.* This project was conducted using UK Biobank application no. 15825. UK Biobank was established by the Wellcome Trust medical charity, Medical Research Council, Department of Health, Scottish Government and the Northwest Regional Development Agency. It has also had funding from the Welsh Government, British Heart Foundation, Cancer Research UK and Diabetes UK. UK Biobank is supported by the National Health Service (NHS). UK Biobank is open to bona fide researchers anywhere in the world.

## Supplementary Material

Web_Material_kwae143

## Data Availability

All the data used in this study are publicly available.
