# Supplementary material for: Comparison of caffeine consumption behavior with plasma caffeine levels as exposure measures in drug-target mendelian randomization
Source: Am J Epidemiol. 2024 Jun 20;193(12):1776–84. doi: 10.1093/aje/kwae143 (PMC7616520; doi:10.1093/aje/kwae143)
Supplement: Web_Material_kwae143 [file web_material_kwae143.zip › supplementary_material.docx]

**Supplementary Material**

**Title:** Comparison of caffeine consumption behavior with plasma caffeine levels as exposure measures in drug-target Mendelian randomization.

**Authors:** Benjamin Woolf, Héléne T. Cronjé, Loukas Zagkos, Susanna C. Larsson, Dipender Gill, Stephen Burgess

**Appendix 1:** Box Explanation of two-way fixed effects.

**Appendix 2:** Supplementary Results

**Appendix 1: Box Explanation of two-way fixed effects.**

Two-way fixed effects (TWFE) is highly popular in the sociology literature (51) and follows similar logic to monozygotic twin difference study designs used in epidemiology (52). A monozygotic twin difference design matches an individual with their monozygotic twin who has a discordant exposure status. Given that these twins generally shared a developmental environment and possess identical genetics, this matching should remove much of the confounding bias related to those exposures. Fixed effects (FE) panel regression leverages multiple observations on the same individual at different times to essentially match an individual with themselves at a different time, thereby controlling for all time-invariant confounding like genetics. The name derives from the fact that the time-invariant portion of the exposure and outcome is controlled for in the model by estimating it as the fixed effect of the variable’s time series. Time-varying confounding is therefore not adjusted for in a traditional FE analysis. However, when data is available from many time points, it is possible to introduce a FE for time in a TWFE analysis (29). This will adjust for linear time-varying confounding (53,54). More detailed descriptions of FE and TWFE can be found elsewhere (26–29,51,55,56). Although assuming only linear time-varying confounding is not always plausible, this assumption is different from the “no pleiotropy” assumption made by MR, and therefore makes TWFE a suitable method to triangulate with MR.

**Appendix 2:** **Supplementary Results**

**Replication of coffee consumption results using tea consumption**

We were able to broadly replicate the results of our coffee consumption-based MR models reported in the main text using the UK Biobank GWAS of tea consumption. We found that each standard deviation (SD) increase in the genetically-predicted amount of self-reported tea consumed was associated with 0.225 (95% CI: 0.045 to 0.405) SD higher BMI. This analysis had variant-exposure F statistic of 104.

Steiger filtering on the variants associated with caffeine consumption behavior implied that 10 of the 26 tea consumption-associated SNPs are affecting caffeine consumption behavior because of their effect on caffeine metabolism. As with the coffee consumption-based analysis, the SNPs which Steiger filtered for caffeine plasma levels imply a negative MR association between circulating plasma caffeine levels and tea consumption (beta = -0.320, 95% CI = -0.465 to 0.175). Again, when using these SNPs to estimate the effect of tea consumption on BMI, we observe a positive MR estimate (beta = 0.256, 95% CI = 0.146 to 0.366), but scaling by plasma caffeine levels result in MR estimates similar to those observed in the biologically-motivated MR analysis (beta = -0.064, 95% CI = -0.131 to 0.003).

The MR analysis of caffeine consumption on BMI using SNPs which Steiger filter for tea consumption still implies that increased tea consumption may increase BMI (beta = 0.150, 95% CI = -0.254 to 0.554). This cannot be explained by the effect these SNPs have on circulating caffeine since the MR of tea-derived caffeine consumption on plasma caffeine levels are indicative of a positive direction of effect (beta = 0.026, 95% CI = -0.239 to 0.291).

Our MVMR model confirmed a direct effect of caffeine consumption , independent of plasma caffeine levels, on BMI. Each SD increase in tea consumption results in a 0.280 (95% CI: 0.073 to 0.568) SD BMI increase independent of plasma caffeine levels. The conditional F statistics for caffeine plasma levels and tea consumption were 4 and 8 respectively.

**References**

51. Hill TD, Davis AP, Roos JM, et al. Limitations of fixed-effects models for panel data. *Socio Perspec*. 2020;63(3):357-369. https://doi.org/10.1177/0731121419863785

52. Angrist JD, Pischke JS. *Mostly Harmless Econometrics: An Empiricist’s Companion*. Princeton University Press; 2009:392.

53. Imai K, Kim IS. On the use of two-way fixed effects regression models for causal inference with panel data. *Politic Anal*. 2021;29(3):405-415. https://doi.org/10.1017/pan.2020.33

54. Jakiela P. Simple Diagnostics for Two-Way Fixed Effects [Internet] arXiv; 2021 2023.Available from: <http://arxiv.org/abs/2103.13229>

55. Imai K, Kim IS. When should we use unit fixed effects regression models for causal inference with longitudinal data? *Am J Polit Sci*. 2019;63(2):467-490. https://doi.org/10.1111/ajps.12417

56. Collischon M, Eberl A. Let’s talk about fixed effects: Let’s talk about all the good things and the bad things. *Köln Z Soziol*. 2020;72(2):289-299. https://doi.org/10.1007/s11577-020-00699-8
