# Supplementary material for: Comparison of caffeine consumption behavior with plasma caffeine levels as exposure measures in drug-target Mendelian randomization
Source: Am J Epidemiol. Author manuscript; Available in PMC 2024 Sep 20. (PMC7616520; doi:10.1093/aje/kwae143)
Supplement: Supplementary file 1 [file EMS197045-supplement-Appendix_1.docx]

Appendix 1: Box Explanation of two-way Fixed Effects. 

Two-way Fixed Effects (TWFE) is highly popular in the sociology literature (51), and has a similar logic to monozygotic twin difference designs used in epidemiology (52). A monozygotic twin difference design matches an individual with their monozygotic twin who has a discordant exposure status. Given that these twins generally share a developmental environment and possess identical genetics, this matching should remove much confounding bias. Fixed effects (FE) panel regression leverages multiple observations on the same individual at different times to essentially match an individual with themselves at a different time, and therefore control for all time-invariant confounding like genetics. The name derives from the fact that the time-invariant portion of the exposure and outcome is controlled for in the model by estimating it as the fixed effect of the variable’s time series. Time-varying confounding is therefore not adjusted for in a traditional FE analysis. However, when data is available from many time points, it is possible to introduce a fixed effect for time in a TWFE analysis (29). This will adjust for linear time-varying confounding (53,54). More detailed descriptions of FE and TWFE can be found elsewhere (26–29,51,55,56). Although assuming only linear time-varying confounding is not always plausible, this assumption is different from the “no pleiotropy” assumption made by MR, and therefore makes TWFE suitable to triangulate with MR.
