# Supplementary material for: Comparison of caffeine consumption behavior with plasma caffeine levels as exposure measures in drug-target Mendelian randomization
Source: Am J Epidemiol. Author manuscript; Available in PMC 2024 Sep 20. (PMC7616520; doi:10.1093/aje/kwae143)
Supplement: Supplementary file 2 [file EMS197045-supplement-Appendix_2.docx]

**Appendix 2:** **Supplementary Results**

**Replication of coffee consumption results using tea consumption** 

We were able to broadly replicate the coffee consumption findings in the main text using the UK Biobank GWAS of tea consumption. We found, in our MR using self-reported tea consumption, that each standard deviation (SD) increase in the genetically-predicted amount of tea consumed was associated with 0.225 (95% CI: 0.045 to 0.405) SD higher BMI. This analysis had variant-exposure F statistic of 104. 

Steiger filtering on the variants associated with caffeine consumption behavior implied that 10 of the 26 tea consumption-associated SNPs are affecting caffeine consumption behavior because of their effect on caffeine metabolism.  As with the coffee analysis, the SNPs which Steiger filtered for caffeine plasma levels imply a negative MR association between circulating plasma caffeine levels and tea consumption (beta = -0.320, 95% CI = -0.465 to 0.175). Again, when using these SNPs to estimate the effect of tea consumption on BMI produces a positive MR estimate (beta = 0.256, 95% CI = 0.146 to 0.366), but scaling by plasma caffeine levels result in similar MR estimates (beta = -0.064, 95% CI = -0.131 to 0.003) to those observed in the biologically-motivated MR analysis. 

The MR analysis of tea consumption on BMI using SNPs which Steiger filter for tea consumption still implies that increased tea consumption may increase BMI (beta = 0.150, 95% CI = -0.254 to 0.554). This again cannot be explained by the effect these SNPs have on circulating caffeine: MR of tea consumption on plasma caffeine levels are indicative of a positive direction of effect (beta = 0.026, 95% CI =  -0.239 to 0.291). 

Our MVMR model again finds some evidence of a direct effect of tea consumption, independent of plasma caffeine levels, on BMI: Each SD increase in tea consumption results in a 0.280 (95% CI: 0.073 to 0.568) SD increase in BMI independent of plasma caffeine levels. The conditional F statistics for caffeine plasma levels and tea consumption were 4 and 8 respectively.
